# Clinical Notes on Obstetric Surgery

**Published:** 1897-07

**Authors:** Joseph Eve Allen

**Affiliations:** Augusta, Ga., Professor of Obstetrics and Pediatrics in the Medical Department of the University of Georgia; Consulting Obstetrician to the City and Lamar Hospitals


ATLANTA

### Medical and Surgical Journal.

VOL. XIV. JULY, 1897. No. 5.

L. B. GRANDY, M.D., MANAGING EDITOR.

M. B. HUTCHINS, M.D., BUSINESS MANAGER.

COLLABORATORS:
A. W. CALHOUN, M.D., LL.D., VIRGIL O. HARDON, M.D., FLOYD W. McRAE, M.D.,
A. W. STIRLING, M.D., JOSEPH EVE ALLEN, M.D., AUGUSTA, GA., AND
HENRY R. SLACK, PH.M., M.D., LAGRANGE, GA.

### ORIGINAL COMMUNICATIONS.

### CLINICAL NOTES ON OBSTETRIC SURGERY.

BY JOSEPH EVE ALLEN, M.D., AUGUSTA, GA.,
Professor of Obstetrics and Pediatrics in the Medical Department of the University of Georgia; Consulting Obstetrician to the City and Lamar Hospitals.

In the following paper are presented the results of a careful study, with reference to operative procedures, of the records of 3,500 cases of labor, that have come under my observation in hospital and private practice during the past twenty-three years:

*The Forceps.*—The forceps were used with primiparæ in fifteen per cent. of the cases and in five per cent. of the multiparæ, uterine inertia being the principal indication for their employment. The causes of inertia in these cases were generally constitutional debility, rigidity of os or perineum, permature rupture of the membranes, large head, or minor pelvic construction. Hospital patients required instrumental delivery much more frequently than those in private practice, owing to their defective stamina and badly rundown condition. The high forceps operation was seldom necessary, the instruments being used generally when the head was in the pelvic cavity, or at the inferior strait.

The forceps I prefer is a combination of the instruments of Barnes and Simpson, having the blades of Barnes and the lock and handles of Simpson. This instrument is easy to adjust, and does not slip or compress the head injuriously. I always apply the blades in accordance with the teachings of the English school, that is along the sides of the pelvis, without any special attempt to grasp the head in its biparietal diameter. The position for delivery was in every case the dorsal decubitus, and the forceps were always removed before the head passed the vulva. Axis traction instruments were only employed when the head was high in the pelvis or above the superior strait, and then either Lusk's axis traction forceps, or Reynold's traction rods were used. In the low operation the ordinary long forceps will subserve the purpose much better than the axis traction, or the short forceps. Short forceps should never be used, because their construction is weak and faulty, and they are difficult to manage, and hence dangerous. The forceps were in these cases not once used to effect rotation; compression of the head was never continuous, but pendulum movements were often resorted to with good effect. The so-called dynamic power of the forceps, that is, their effect by reflex action in increasing the force and efficiency of the uterine contractions, was often noted. This dynamic power of the forceps should be ever borne in mind in operating, especially on multiparæ, for if forgotten extensive laceration of the soft parts may be the result.

*Version.*—Version was required in this series of cases in multiparæ about as often as in primiparæ, viz.: in three per cent. External version was more frequently performed in primiparæ, and internal in multiparæ. Hick's bipolar method was seldom resorted to. In doing podalic version for transverse presentation, I have found that it is always a matter of great consequence which foot is brought down, notwithstanding what obstetric writers have said to the contrary. When the fetal back is directed anteriorly, the lower foot is the one to turn by, and when posteriorly, the upper foot should be extracted first. This rotates the child's body on its long axis and brings the face into the hollow of the sacrum, which is the most favorable position for the delivery of the aftercoming head. The position for performing version was always with the woman on her back.

*Induction of Premature Labor.*—The premature termination of gestation was more often necessary in cases of women who had had children than in those pregnant for the first time. The indications for this operation were antepartum hemorrhage, due to placenta previa, or accidental separation of the placenta, uremia, septicemia, pernicious nausea and vomiting, and valvular lesions of the heart. It was never done on account of excessive pelvic deformity, because our southern women are so well formed that cases are exceedingly rare in this section demanding this operation.

Aseptic technique has made the induction of premature labor a perfectly safe procedure, and it is best in most cases to complete the dilatation of the os and the emptying of the uterus at one sitting.

*Embryotomy.*—Cutting operations upon the fetus were necessary about once in 700 cases, and were never done in any case where the child was alive. It is my opinion that with the means of delivery that science now places at the disposal of the obstetrician, sacrificial operations on the living child are never justifiable. The instrument generally used to reduce the size of the fetal head was Baxton Hicks's cephalotribe, which was always applied without first perforating. This method has the advantage of keeping the sharp spiculæ of bone within the scalp, and thus preventing wounds of the genital tract. In my experience, the perforator and cranioclast are rarely called for. These instruments are chiefly valuable in cases of great pelvic contraction. Ramsbothom's decapitating knife is, I think, a much more useful instrument in cases of impacted transverse presentation than Braun's hook. Evisceration was only twice required, once in a case of breech presentation with fetal ascites, and once in a case of impacted cross-birth, the child being the second of triplets.

*Hemorrhage.*—Accidental hemorrhage before delivery was treated by rapid dilatation and version. In every case this practice was successful in saving the life of the mother, except one who was moribund when first seen. The children were all stillborn beyond resuscitation. Placenta previa, when partial, was treated by vaginal tampon, rapid instrumental and manual dilatation, separation of placenta from lower uterine zone, artificial rupture of membranes, forceps, or version. Complete placenta previa was treated by vaginal tampon, followed by rapid stretching of the cervix and podalic version. In these cases turning was effected by passing the hand directly through the placental mass. The maternal mortality in cases of complete placenta previa was five per cent.; the infantile mortality was sixty per cent. Whenever the diagnosis of placenta previa is certain, the best interests of mother and child demand the immediate bringing on of labor under aseptic precautions.

Postpartum hemorrhage required treatment in fifteen per cent. of the cases. Two cases of primary postpartum hemorrhage were fatal; one was a woman in the hands of a midwife, who was pulseless and dying when I was called in, the other was a young woman in the last stages of pulmonary consumption, who died one hour after the delivery from shock due to the loss of what, under other circumstances, would have been a very moderate amount of blood. The treatment that in the majority of cases was sufficient to control the flooding was an intrauterine douche of sterilized water at the temperature of 112 degrees Fahrenheit. Tincture of iodine one part, and hot water six or eight parts (Trask's formula), was beneficial in some cases that did not yield to the hot water douche alone. In one instance the use of vinegar produced firm contraction after hot water, tincture of iodine, and other remedies had failed. I have seldom used the iron salts locally to arrest postpartum hemorrhage, for the reason that, as these agents act only by plugging up the uterine sinuses, I believe that they are extremely dangerous, and should not be employed as long as there is any hope of exciting the womb to action by other means. Ergot, whether given by the mouth or hypodermically, has proven of no value whatever, and is not to be depended upon. The drug that is of the most benefit in flooding is strychnia, administered in large doses by puncture. It tetanizes the uterus, and by its tonic action on the heart and nervous system combats shock and depression.

Secondary postpartum hemorrhage occurred in ten cases, with one fatal result, the woman dying on the fifth day after a very difficult forceps delivery. This patient had eclampsia during labor and was hydremic and extremely debilitated. The hemorrhage, which was due to uterine relaxation, occurred suddenly, and she died before I reached her. The treatment of excessive bleeding coming in two or three days after delivery of course depends upon the exciting cause. When produced by a flabby condition of the uterus, intrauterine douches of hot sterilized water, together with packing the cavity with absorbent cotton, saturated with tincture of iodine, generally had the most happy effect. When the flooding was due to retained secundines, the douche and finger accomplished all that was necessary and the curette was never required. Bleeding from the circular artery in two cases of cervical laceration was stopped by pressure with pledgets of gauze soaked in Monsel's solution. When collapse from loss of blood is imminent in lying-in patients,the subcutaneous injection of normal salt solution, by means of an Allen pump, together with high rectal injection of whisky and water, are equally as beneficial as intravenous transfusion.

*Perineorrhaphy.*—Lacerations of the perineum, whether through the sphincter ani or not, were always closed at once, and union by first intention resulted in about thirty per cent. of all cases, including complete and incomplete ruptures. When the tear extended through the rectum, the gut wall was brought together with silk sutures, and the perineum with a buried suture of chromatized catgut, or interrupted sutures of silkworm gut. The practice of keeping the bowels open is decidedly preferable to that of locking them up with opium, which was the old rule, as it is followed by a much larger percentage of cures. Episiotomy was rarely performed, as it appeared to have no effect in the prevention of laceration.

*Septicemia.*—The practice of giving medicines for the cure of infection during childbed has long since become a thing of the past among scientific obstetricians. This condition is now treated upon just the same principle as when it occurs after wounds and surgical operations. It is interesting to compare the results of practice before and after the adoption of antisepsis. When cases were not treated by antiseptic methods, the mortality was from six to ten per cent., and the morbidity from fifteen to twenty per cent.; now the mortality is practically nothing, and the morbidity seldom reaches eight or ten per cent. The method of treatment that I have found to be best in puerperal septicemia is first to make the genital tract thoroughly aseptic, and then sustain the patient until the emunctories of her system can eliminate the poison that has been absorbed. The puerperal uterus should not be curetted, as this only opens up new avenues of infection. An intrauterine douche of creolin, followed by a loose packing of iodoform gauze for drainage, if done early, almost always results in a fall of temperature and relief of other symptoms. When the patient is seen late in the course of the disease, at a time when the structures around the womb have become invaded by colonies of pathogenic germs, and the entire system profoundly poisoned and depressed, the line of treatment is to stimulate and build up the flagging vital forces. In connection with local disinfection, the agent that best subserves this purpose is strychnia, given in large doses hypodermically. Alcoholic stimulants and predigested foods are also required. Large doses of quinine (twenty to thirty grains) seem, in some cases, to have a directly antidotal effect. Morphia should be avoided, as it greatly increases the symptoms of toxemia.

*Milk Fever.*—An investigation of puerperal temperatures in the Maternity Department of the Augusta City Hospital convinces me conclusively that when the labor has been managed aseptically a rise of temperature never takes place at the time of the establishment of the lacteal secretion. In the last twenty-five deliveries that took place in this institution the temperature in no case exceeded 100 degrees Fahrenheit. Milk fever, in my opinion, is a mild form of infection, starting usually from the intestinal tract, and is effectually prevented by cleanliness during delivery and the use of saline cathartics (Epsom salts) afterwards.

*The Asepsis of the Umbilical Stump.*—The careful dressing of the umbiblical stump is a duty that is not beneath the dignity of the obstetric surgeon and should never be left to untrained nurses and old grannies, because it is just as important to protect the infant as the mother, and it is through the navel that the infantile system is generally infected. Since the adoption of the method of dressing the cord described by me in the *American Journal of Obstetrics*, vol. XXIX., No. 4, I have not had in my own practice a single case of tetanus, erysipelas, or septicemia in a newborn infant. The following is this method, viz.:

1st. The cord is secured by clamp or ligature about two and a half inches from abdomen. The child is then turned over to the nurse to be washed.

2d. The physician, having made his hands aseptic, proceeds to cleanse the stump and surface of the abdomen of the child with tincture of green soap and water and afterwards with a 1 to 1,000 bichloride solution.

3. The cord is then again cut and stripped of its gelatinous material and retied with a sterilized silk elastic ligature. The bichloride solution is again used and the stump enveloped in a pad of iodoform gauze soaked in chemically pure glycerine. This dressing is held in place by a sterilized flannel bandage.

(*To be continued.*)

---

### A GRANDMOTHER AT TWENTY-SIX YEARS.

Elizabeth S., a native of Wayne county, N. C., bore an illegitimate daughter in May and reached her thirteenth birthday the following June. The daughter, Ann, menstruated at eleven years, was married at twelve, and bore a son in April, becoming thirteen years old in May. The labor was tedious and difficult. Ann grew four inches taller after bearing this child,and has borne seven children since. She is in fair health and does not show any signs of premature age. Who can beat this record?—*Dr. H. O. Hyatt, Philadelphia Polyclinic.*

